# Electrospun Amorphous Indium Gallium Zinc Oxide (IGZO) Nanofibers for Highly Selective H_2_S Gas Sensing

**DOI:** 10.3390/s26061936

**Published:** 2026-03-19

**Authors:** Anh-Duy Nguyen, Sung Tae Lim, Jong Heon Kim, Yujin Kim, Gayoung Yoon, Ali Mirzaei, Hyoun Woo Kim, Sang Sub Kim

**Affiliations:** 1 Department of Materials Science and Engineering, Inha University, Incheon 22212, Republic of Korea; 2 Program in Biomedial Science and Engineering, Inha University, Incheon 22212, Republic of Korea; 3 Department of Materials Science and Engineering, Shiraz University of Technology, Shiraz 715557-13876, Iran; mirzaei@sutech.ac.ir; 4 Division of Materials Science and Engineering, Hanyang University, Seoul 04763, Republic of Korea; hyounwoo@hanyang.ac.kr

**Keywords:** IGZO, electrospinning, nanofiber, H_2_S, gas sensor, sensing mechanism

## Abstract

As a ternary metal oxide, indium gallium zinc oxide (IGZO) has gathered much attention for various applications, including gas sensors, due to its remarkable semiconducting properties, even in amorphous phases and at a low process temperature. For gas sensing applications, as surface area is an important factor affecting the response and performance of a gas sensor, nanofibers (NFs) with 1D morphology are expected to have good sensing performance. In this research, IGZO NFs were synthesized using an electrospinning process, which is a suitable technique for the large-scale and low-cost fabrication of NFs. Various characterizations were performed on the synthesized IGZO NFs, and the desired NF morphology and chemical composition were confirmed. Gas sensing experiments showed that the sensor was sensitive and selective to H_2_S gas at 250 °C with a response of 40.5 to 100 ppm gas. This study demonstrates the strong potential of IGZO for use in sensitive and selective H_2_S gas sensors.

## 1. Introduction

H_2_S is known to be a highly toxic, flammable, and corrosive gas with a distinct rotten egg odor. It is emitted from various activities such as mining, excavation, or industrial processing of crude oil and natural gas, as well as animal waste processes [1]. H_2_S exposure can have severe impacts on human health, ranging from irritated eyes, nose, and respiratory system at low concentrations to fatal consequences at concentrations of 100 ppm or above [2]. Moreover, it is also a highly corrosive agent, causing the failure of pipelines and equipment [3]. Furthermore, H_2_S can also serve as a biomarker for the diagnosis of halitosis [4] and other diseases. Accordingly, H_2_S detection is vital in different aspects. To detect H_2_S gas, various gas sensors, including but not limited to optical [5], acoustic wave [6], thermoelectric [7], electrochemical [8], calorimetric [9], and resistive [10], have been developed. Each type of sensor offers advantages and drawbacks. This limits their applications in certain situations. Among them, metal oxide resistive sensors are known for high sensitivity, stability, fast response, simple design, and low costs [11]. However, they also possess some disadvantages, such as a high operating temperature and poor selectivity [12]. The high sensing temperature limits their applications in low-power and portable devices or in remote areas, where energy access is limited, while poor selectivity may cause false alarms in real applications. The requirements for high operating temperatures usually stem from the adsorption and activation energy of the gas species [13]. These play important roles in sensor sensitivity and response times, which are critical to sensor performance. However, the heating process can result in degradation and reduced long-term stability due to the alteration of granular structures [14]. Furthermore, high thermal budget can translate to higher fabrication costs. To address this issue, multiple strategies have been developed and studied, such as noble metal incorporation [15,16] and built-in junctions [17]. Another potential approach is integrating a dual heterojunction, which was shown to be effective in enhancing the sensitivity of zeolitic imidazolate framework-8 [18]. Because of the mentioned reasons, metal oxide materials have been gathering a lot of research momentum.

Since its development in 2004, indium gallium zinc oxide (IGZO) has been gathering intensive research attention [19]. IGZO has become one of the best-known demonstrations of a complex amorphous oxide [20]. It has low synthesis costs, non-toxicity, and high electron mobility (>10 cm^2^/V·s), as well as compatibility with the semiconductor industry [21]. This makes IGZO an interesting and popular choice in different applications, ranging from flexible devices and transparent electronic devices to next-generation devices for neuromorphic computing [22]. One of the major advantages of IGZO is that it can achieve the above properties while remaining in an amorphous phase [23], resulting in high performance and semiconducting characteristics without annealing at high temperatures. This circumvents the requirement of demanding thermal treatment, which opens up the possibility of utilizing the material for various applications. Hence, amorphous IGZO (a-IGZO) has been extensively studied for gas sensing applications. For example, a-IGZO thin film gas sensors with strong performance were demonstrated for NO_2_ at 200 °C and for H_2_ detection at 350 °C by Yang et al. [24]. Also, Jaisutti et al. [25] reported enhanced acetone sensing of a-IGZO at room temperature in the presence of UV light. Huang et al. [26] reported a highly selective H_2_ gas sensor based on ZIF-8 coating.

To improve the gas sensing performance of resistive gas sensors, various approaches have been implemented. Functionalization and loading with other materials have been reported to significantly improve sensing performance [27]. Also, plasma irradiation can effectively improve or modify the surface and increase the sensitivity of sensing materials [28]. Morphology engineering is another effective method to improve sensing response through increasing the surface area. In this context, nanofibers (NFs) offer a large surface area for target gas molecules [29]. Furthermore, the composition and characteristics of NFs can be controlled to achieve the desired properties [30]. These features make NFs attractive for gas sensing applications. Electrospinning is known for its ability to fabricate NFs on a large scale while maintaining good quality and flexibility in the process [31]. Because of these reasons, electrospun NFs are attractive for gas sensing applications.

In this research, a-IGZO NFs were synthesized through electrospinning. The target of this study was to utilize the advantages provided by the amorphous phase of IGZO and further improve its gas sensing performance through the large surface area of NFs’ morphology. Furthermore, this work also aims to highlight a low-cost fabrication route with a relatively low thermal budget. The amorphous nature of the NFs was confirmed through XRD and TEM analyses. The sensor was exposed to various gases, and at 250 °C, it showed the maximum response of 40.5 to 100 ppm H_2_S gas. Moreover, the sensor demonstrated excellent selectivity toward H_2_S. The fabricated amorphous material exhibited stability and consistent performance throughout the testing process. The underlying sensing mechanisms are discussed in detail.

## 2. Experimental Procedure

### 2.1. Chemicals

Analytical-grade indium(III) nitrate hydrate (In(NO_3_)_3_xH_2_O), gallium nitrate hydrate (Ga(NO_3_)_3_xH_2_O), zinc nitrate hydrate (Zn(NO_3_)_3_xH_2_O), and N,N-Dimethylformamide (DMF), as well as polyvinyl alcohol (PVA; MW ~80,000), were obtained from Merck KGaA, Darmstadt, Germany.

### 2.2. Synthesis of a-IGZO NFs

**Figure 1:**
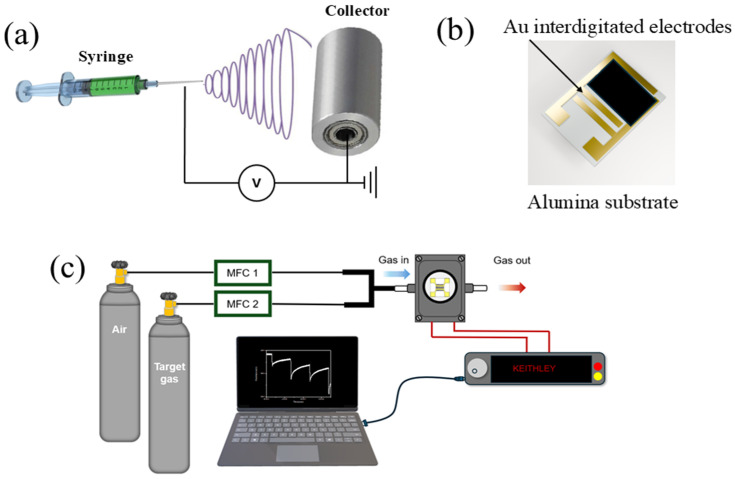
Schematic of (**a**) electrospinning process, (**b**) fabricated gas sensor, and (**c**) gas sensing measurement system.

First, 10 wt% PVA was dissolved into a mixture of 20 mL of ethyl alcohol and DMF with a volume ratio of 1:1. Subsequently, a mixture of In(NO_3_)_3_xH_2_O (0.176 g), Ga(NO_3_)_3_xH_2_O, (0.150), and Zn(NO_3_)_3_xH_2_O (0.174 g) was added to the above solution and stirred for 2 h to achieve a viscous solution suitable for electrospinning. The weights of precursors were controlled so that the final In, Ga, and Zn composition ratio was 1:1:1. The viscous solution was then put into a plastic syringe (1.13 mm in diameter), which was mounted on an anode linked to a high-voltage power supply. Also, the cathode was a collector at a fixed distance of 14 cm from the anode. For the electrospinning process, a 16 kV voltage was applied, and the distance between the needle and the collector was fixed at 14 cm. Upon applying a 16 kV voltage and a feed rate of 0.5 mL/h, IGZO NFs started to form (Figure 1a). Generally, the crystallization temperature of IGZO is higher than 500 °C [2,24,32]. The stability of the amorphous phase at high annealing temperatures can be attributed to the incorporation of indium, gallium, and zinc cations in a-IGZO. The existence of cations with different ionic charges and sizes is beneficial for improving the formation of an amorphous phase and subduing crystallization [33]. The stability of the amorphous phase is important to prevent partial or complete crystallization in the network, as undesired grain growth can affect the thermal stability of the sensors [14]. To maintain the amorphous nature of the electrospun NFs, and to effectively remove the organic species on the IGZO NFs without initiating IGZO crystallization, they were heat-treated at 500 °C/2 h in air. To confirm the successful formation of IGZO nanofibers, a separate sample was heat-treated at 800 °C/2 h in air to achieve high crystallinity. Furthermore, a sample annealed at 400 °C/2 h in air was also prepared to provide a reference for organic residual analysis in the XPS results.

### 2.3. Material Characterization

The formation of a-IGZO NFs and their morphologies were analyzed through field emission scanning electron microscopy (FE-SEM; Hitachi S-4200, Tokyo, Japan) and transmission electron microscopy (TEM; JEOL; Tokyo, Japan). Energy-dispersive X-ray spectroscopy (EDS) incorporated in TEM was utilized to determine the chemical composition. The amorphous nature of a-IGZO NFs was analyzed using X-ray diffraction (XRD; Philips X’Pert, Almelo, The Netherlands). Finally, the surface chemical states of the NFs were studied by X-ray photoelectron spectroscopy (XPS; Thermo Scientific, Waltham, MA, USA). All XPS spectra were calibrated to the adventitious carbon C 1s peak at 284.8 eV. This value was selected because it is the most widely accepted calibration standard for hydrocarbon contamination present on air-exposed surfaces such as MXenes. Also, all spectra were background-corrected prior to peak fitting using a Shirley background, which was selected as appropriate for the narrow energy windows and core-level regions analyzed. Regarding the XPS fitting, we used the following procedures: (i) To account for both instrumental broadening and lifetime broadening, core-level peaks were fitted using mixed Gaussian–Lorentzian line shapes. (ii) Chemically related components were constrained considering (a) fixed spin–orbit splitting values, (b) constrained area ratios, and (c) tied peak positions which were appropriate for chemically equivalent states. (iii) Peaks assigned to the same chemical state were fitted with tied full-width at half maximum (FWHM) values, while different chemical states were permitted limited variation within physically reasonable bounds. (iv) Residual plots (data–fit) are included for all fitted spectra.

### 2.4. Gas Sensing Test

The a-IGZO NFs were mixed with α-terpineol (10 µL), and then they were applied on an alumina substrate with a pre-made golden interdigitated electrode pattern with a comb size of 100 µm (Figure 1b). The fabricated sensor was further dried in air at 350 °C for 2 h. Gas sensing tests were performed using a lab-made sensing system and an MSTECH-MMVC2S chamber (Incheon, Korea) (Figure 1c) with a total volume of 517.5 cm^3^. The resistance of the gas sensor was continuously measured using a Keithley 2450 source meter in the air (R_a_) and in the presence of the target gas (R_g_). The targeted gas concentrations were produced through accurate mass flow controllers (MFCs) with a total gas flow rate set to 500 sccm. The gas response was calculated as R = R_a_/R_g_ and R = R_g_/R_a_ for reducing gases and oxidizing gases, respectively. The response/recovery times were calculated as the time required for the signals to reach to 90% of the final value from the initial value. The gases used for testing have a concentration of 100 ppm with dry air as a balancing gas.

To study the effect of humidity on sensor performance, sensing tests were performed under varied relative humidity (RH) levels ranging from 0% to 90%. Fully saturated air (100% RH) was generated by bubbling dry air through water in a sealed vessel. Specific RH levels (30%, 60%, 90 RH%) were achieved by blending appropriate amounts of humid and dry air using MFCs.

## 3. Results and Discussion

### 3.1. Characterization Studies

**Figure 2:**
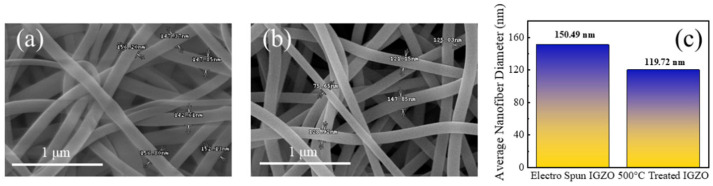
SEM images of (**a**) as-electrospun IGZO NFs and (**b**) heat-treated IGZO; (**c**) average nanofiber diameter for the NFs.

Figure 2a,b provide SEM images of the electrospun NFs before and after heat treatment, respectively. It is obvious that long and continuous NFs were successfully formed. Figure 2c shows the average diameters of the NFs before and after heat treatment, which were 150.49 and 119.72 nm, respectively. The decrease in diameter of the NFs after heat treatment could possibly be attributed to the evaporation of the solvent and the organic species at a high temperature. However, it should be noted that even after heat treatment, some organic additives, even in small quantities, may be present.

**Figure 3:**
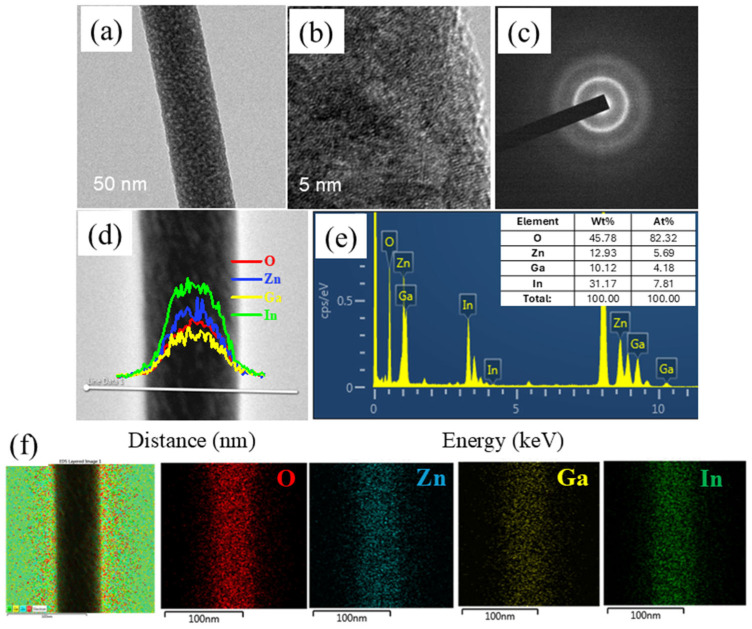
(**a**) TEM and (**b**) HRTEM images of a-IGZO NFs, (**c**) SAED pattern of a-IGZO NFs, (**d**) TEM-EDS line mapping and (**e**) corresponding analysis results of a-IGZO NFs, (**f**) TEM-EDS mapping analysis of a-IGZO NFs.

TEM was further utilized to analyze the morphology of the NFs after heat treatment (Figure 3a). It can be observed that the NFs’ surfaces are smooth, with no formation of a grain structure. Figure 3b provides a high-resolution TEM image of the a-IGZO NFs, in which no crystalline structure can be observed. The SAED pattern illustrated in Figure 3c also confirms the lack of a crystalline nature of the a-IGZO NFs. The distribution of the respective elements along the diameter of a typical NF was explored using TEM-EDS line mapping (Figure 3d). The weight percentages of oxygen, indium, gallium, and zinc were 45.78, 12.93, 10.12, and 31.17 wt%, respectively, and the atomic percentages of oxygen, indium, gallium, and zinc were 82.32, 7.81, 4.18, and 5.69 at%, respectively (Figure 3e). Therefore, the In:Ga:Zn ratio in the observed NF is roughly 1:0.66:0.66. The deviation from the 1:1:1 ratio target could be due to the local fluctuation of the elemental composition in TEM-EDS line mapping. The EDS mapping results are presented in Figure 3f. The uniform presence of oxygen, indium, gallium, and zinc throughout the captured area can be confirmed.

**Figure 4:**
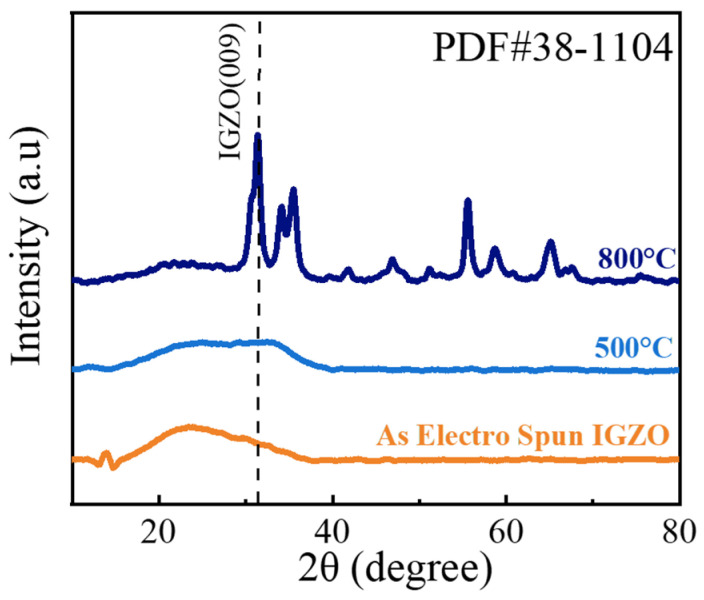
XRD patterns of a-IGZO NFs before and after heat treatment at 500 °C and 800 °C.

Figure 4 presents the XRD patterns of the IGZO NFs before and after heat treatment at 500 °C as well as 800 °C. The XRD pattern of as-synthesized IGZO has a broad peak, reflecting the amorphous nature of IGZO. For the sample heat-treated at 800 °C, strong diffraction peaks can be observed. According to the standard PDF database (PDF#38-1104), the peak with the highest intensity is derived from the (009) plane of crystal IGZO. This confirms that when the heat treatment temperature is sufficiently high, crystalline IGZO NFs can be achieved. The XRD pattern of the sample heat-treated at 500 °C shows a minor peak around 31.1°. However, its intensity is small, and it can be concluded that the sample is still amorphous even after heat treatment at 500 °C. In conclusion, the XRD analysis shows that amorphous IGZO NFs were successfully fabricated.

**Figure 5:**
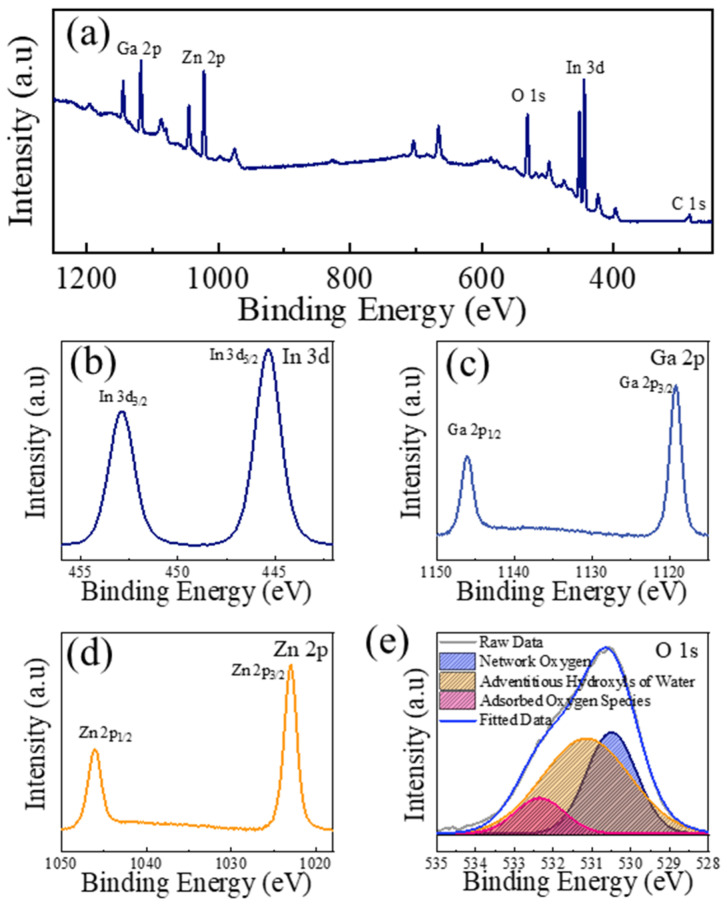
(**a**) XPS survey of a-IGZO NFs. XPS core-level regions of (**b**) In 3d, (**c**) Ga 2p, (**d**) Zn 2p, and (**e**) O1s.

To reliably analyze the chemical composition of the a-IGZO NFs, XPS was utilized for the NFs after heat treatment. First, to confirm the elimination of the organic compounds after annealing at the chosen temperature, the XPS spectra of samples treated at 400 °C, 500 °C, and 800 °C were compared. The spectra can be examined in Figure S1. It can be observed that when the heat-treatment temperature increases from 400 °C to 500 °C, the intensity of the C 1s peak reduces significantly. Furthermore, the C 1s peaks of 500 °C and 800 °C have similar intensities. Therefore, it is reasonable to suspect the effective extermination of organic species at the targeted temperature. Figure 5a shows the XPS survey of IGZO NFs annealed at 500 °C. Figure 5b–d display the XPS core levels of In 3d, Ga 2p, and Zn 2p, respectively. The survey spectrum was calibrated based on the C 1s peak. The core-level data compensate for the background signal. The raw and background-corrected spectra are provided in Figure S2. The deconvoluted O 1s XPS core level is presented in Figure 5e. It comprises three peaks centered at 530.5, 531.15, and 532.35 eV, which can be attributed to network oxygen, adventitious hydroxyls of water, and adsorbed oxygen species, respectively [34,35]. The calculated percentages of the three peaks mentioned were 34.24, 51.94, and 13.80%, respectively. The residual spectrum can be examined in Figure S2d. In conclusion, XPS analysis confirmed the desired chemical composition of IGZO without any impurity. It should be noted that ex situ XPS cannot be used to directly identify oxygen vacancies. However, indirect observations are only feasible if performed in operando and the experiments are appropriately designed [36]. XPS analysis also reveals the elemental composition of the fabricated NFs. Atomic percentages for NFs annealed at different temperatures are provided in Table S5. For NFs annealed at 500°C, the atomic percentages of In, Ga, Zn, and O are 13.02, 15.45, 15.3%, and 56.20%, respectively. Overall, it can be observed that the In:Ga:Zn ratio of 1:1:1 was achieved.

### 3.2. Gas Sensing Studies

**Figure 6:**
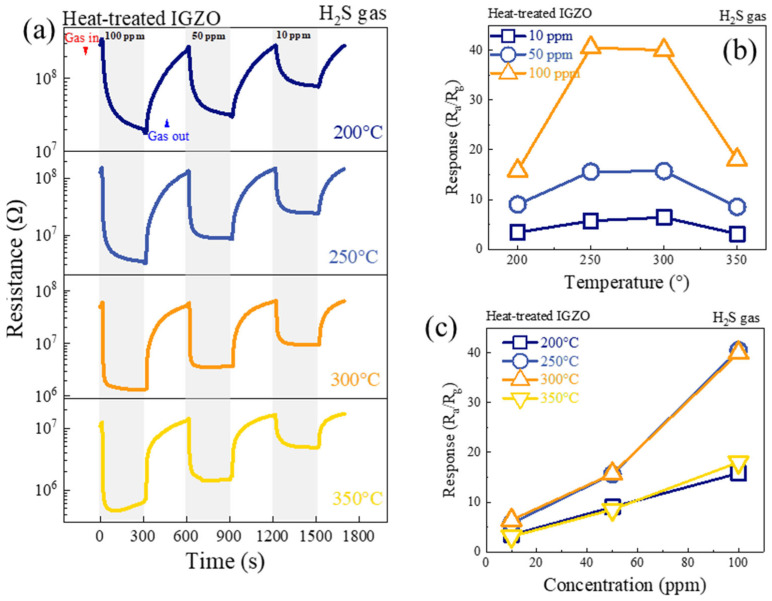
(**a**) Dynamic resistance curves of a-IGZO NF gas sensor to 100, 50, and 10 ppm H_2_S from 200 °C to 350 °C, (**b**) response versus temperature, and (**c**) response versus gas concentration.

To investigate the optimal sensing temperature for the a-IGZO NF gas sensor, it was exposed to 100, 50, and 10 ppm H_2_S gas at different temperatures. Figure 6a provides dynamic sensing curves, with resistance shown on a logarithmic scale. When the sensor was in contact with H_2_S, its resistance sharply decreased, confirming the n-type behavior of a-IGZO NFs, which is consistent with previous studies [22,24,26]. The response was dependent on both gas concentration and sensing temperature (Table S1). To further analyze the effect of temperature as well as gas concentration, the response values of a-IGZO NFs were calculated and plotted against temperature and concentration, as shown in Figure 6b and Figure 6c, respectively. The response showed a maximum at 250 °C, and then it decreased due to the fact that the desorption rate increased relative to the adsorption rate. At 250 °C, the response of the sensor towards 10, 50, and 100 ppm was 5.76, 15.61, and 40.52, respectively. Also, the response increased with increasing gas concentration at all tested temperatures. In fact, with increasing concentration, more gas was adsorbed on the sensor surface, leading to a larger resistance change. To confirm the consistency of the acquired results, four sensors were fabricated by the above method and characterized in the same conditions. The test data is presented in Figure S3. The test results support the reliability of the acquired results. Figure S4 presents the baseline resistance dependency on the temperatures.

**Figure 7:**
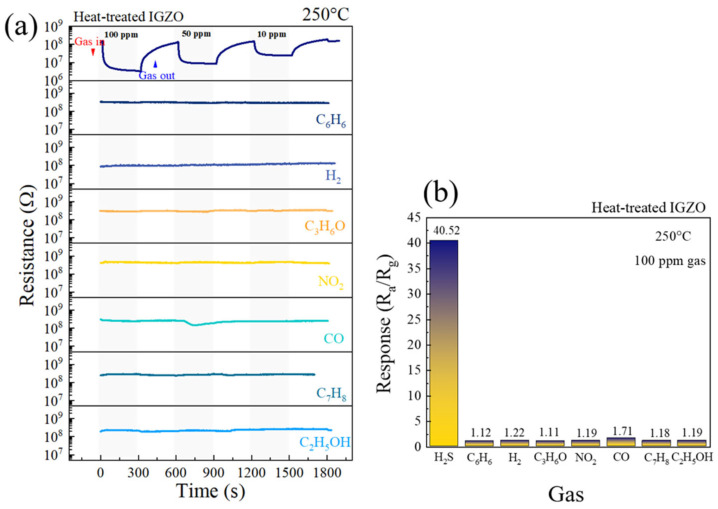
(**a**) Dynamic resistance curves of a-IGZO NF gas sensor to 100, 50, and 10 ppm of various gases at 250 °C and (**b**) corresponding selectivity histogram.

Metal oxide gas sensors usually face challenges regarding selectivity. Therefore, the a-IGZO NFs sensor was exposed to various gases to investigate its selectivity. Dynamic resistance curves for 100, 50, and 10 ppm of different gases (benzene, toluene, hydrogen, acetone, ethanol, carbon monoxide, nitrogen dioxide) at 250 °C and corresponding selectivity histograms are presented in Figure 7a (with resistance shown on a logarithmic scale) and Figure 7b, respectively. The sensor was insensitive to interfering gases, with R_g_/R_a_ or R_a_/R_g_ much less significant in comparison to H_2_S (Table S2). The responses toward H_2_S, C_6_H_6_, H_2_, C_3_H_6_O, NO_2_, CO, C_7_H_8_, and C_2_H_5_OH were 40.52, 1.12, 1.22, 1.11, 1.19, 1.71, 1.18, and 1.19. Thus, the sensor was highly selective to H_2_S gas, which is of importance for real applications.

**Figure 8:**
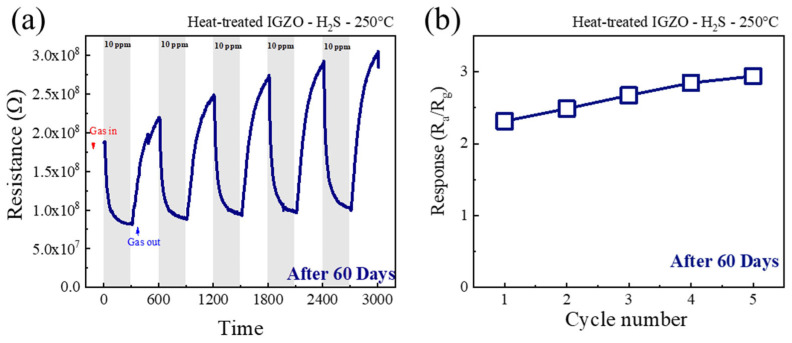
(**a**) Repeatability test of a-IGZO NF gas sensor response after 60 days to 10 ppm H_2_S at 250 °C during five sequential cycles, (**b**) response at different sensing cycles.

To study the long-term stability and repeatability of the a-IGZO NF sensor, 60 days after the fabrication, the sensor was exposed to 10 ppm H_2_S at 250 °C during five continuous cycles (Figure 8a). The corresponding calibration graphs are presented in Figure 8b. The response values were 2.31, 2.48, 2.67, 2.84, and 2.93 during cycles 1 to 5, respectively. In comparison to the initial response to 10 ppm H_2_S (R_a_/R_g_ = 5.76), there is an observable reduction. This reduction can be attributed to the adsorption/desorption of oxygen and water molecules. This phenomenon has been explored in other studies and remains a challenge [37,38]. Nevertheless, the sensor still exhibits reasonable sensitivity and repeatability throughout many cycles after 60 days.

**Figure 9:**
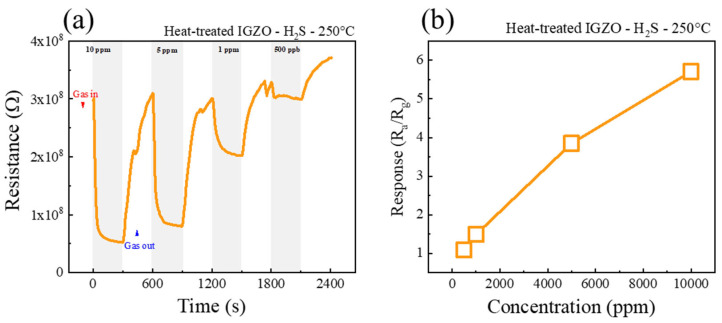
(**a**) Dynamic resistance curves of a-IGZO NF sensor to 10, 5, 1, and 0.5 ppm H_2_S gas at 250 °C, (**b**) corresponding calibration curve.

Next, we studied the response of gas sensors to low concentrations of H_2_S gas at 250 °C, as shown in Figure 9a. Figure 9b shows the corresponding calibration curve. The response values to 10, 5, 1 ppm, and 500 ppb were 5.71, 3.85, 1.48, and 1.09, respectively. Using the current measuring system, the signal-to-noise ratio is around 10%. Therefore, the response toward 500 ppb is too small to reliably assess. As a result, the smallest H_2_S concentration which was experimentally detected was 1 ppm. The response shows a linear dependence on gas concentrations.

**Figure 10:**
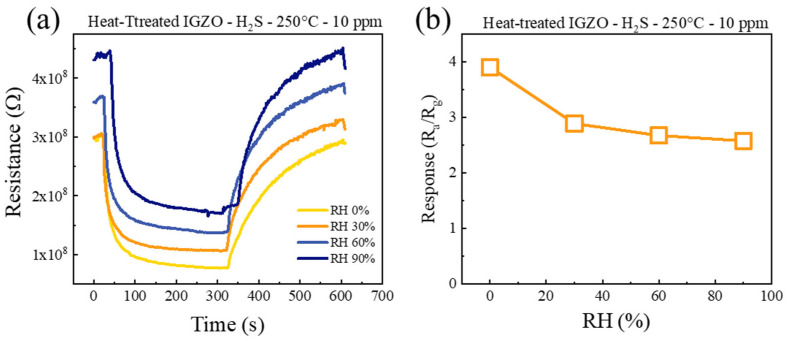
(**a**) Sensing curves of the a-IGZO NF sensor towards 10 ppm H_2_S gas in the presence of various levels of RH at 250 °C, (**b**) response value versus RH.

The impact of humidity on the H_2_S gas sensitivity of an a-IGZO NF sensor was also studied. The fabricated sensor was exposed to 10 ppm H_2_S at 250 °C and different RH levels (0, 30, 60, and 90%) (Figure 10a). Figure 10b presents the corresponding calibration graph. The response values of the sensor in the presence of 0, 30, 60, and 90% RH were 3.89, 2.89, 2.67, and 2.57, respectively. Thus, there is a noticeable reduction in the response value with the increase in humidity level. This is in agreement with previous reports [39,40]. This could seriously limit the integration of the fabricated material into multiple applications. This major challenge will be addressed in future work. The degradation could be attributed to the competition between the H_2_S gas and water molecules to occupy the adsorption sites. Water molecules are considered to be one of the most harmful factors for semiconductor gas sensors. In the range of 20–80% RH levels, the water molecule concentration can reach 14,000–56,000 ppm at 38 °C, significantly higher than the target gas. Furthermore, in the presence of water molecules, some adsorption sites are occupied by them and, hence, the number of adsorption sites for H_2_S gas molecules decreases, leading to a lower response in humid environments relative to dry conditions [41]. The adsorption of water leads to the formation of hydroxyl groups. This in turn blocks oxygen adsorption on the surface, which is crucial for gas sensitivity [42]. Furthermore, it has been reported that water can also inhibit sensing through blocking oxygen vacancies in semiconductors [43] and reduce the affinity of the adsorbed species toward the target gas [44]. The variation in environmental humidity also causes an alteration in the baseline resistance and reduces the long-term stability of the sensor. Humidity has been singled out as one major factor impacting the performance of IGZO devices. Studies on the degradation of the electrical properties of IGZO caused by humidity have been conducted [45,46], but humidity resistance for IGZO still requires further investigation and improvement. Potential approaches include the incorporation of protective layers such as ultra-thin SiO_2_ films [47] and hydrophobic zirconia films [48], or the compensation of humidity-induced drift through the development of software [49].

Table 1 compares the H_2_S gas sensing performance of the present a-IGZO NF sensor with that of other sensors reported in the literature. Overall, the sensor shows a higher response relative to the listed gas sensors, demonstrating the strong potential of a-IGZO for H_2_S gas sensing.

### 3.3. Gas Sensing Mechanism

**Figure 11:**
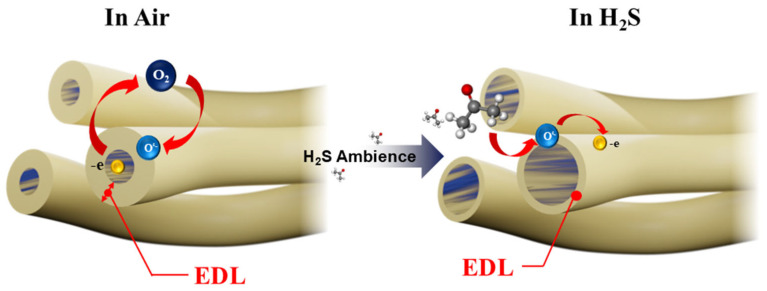
H_2_S gas sensing mechanism of a-IGZO NFs.

Figure 11 shows a schematic of the sensing mechanism of the present gas sensor. The basic sensing mechanism of a resistive gas sensor is based on the modification of its electrical resistance when the material is introduced to a target gas. In clean air, oxygen molecules are adsorbed on the a-IGZO NFs’ surface. Because of the high electron affinity of oxygen, these molecules capture electrons from the sensor surface as follows:

(1) $$O_{2}(g)\to O_{2}(ads)$$

(2) $$O_{2}\left(ads\right)+e^{-}\to O_{2}^{-}\left(ads\right)$$

(3) $$O_{2}^{-}\left(ads\right)+e^{-}\to {2O}^{-}\left(ads\right)$$

(4) $$O^{-}+e^{-}\to O^{2-}\left(ads\right)$$

Due to the abstraction of electrons, an electron depletion layer (EDL) is formed on the surface of the a-IGZO NFs. In this layer, the electron concentration is lower compared to the interior of the NFs. Therefore, the NFs’ resistance in clean air is considerably higher than in vacuum conditions, where there are no adsorbed oxygen species on the sensor surface. As H_2_S is introduced into the gas chamber, the pre-adsorbed oxygen species start to react with the H_2_S gas. As a result, electrons are released on the sensor surface, and the EDL thickness reduces, leading to decreases in electrical resistance and the appearance of a sensing signal. The relevant reactions are presented as follows:

(5) $$H_{2}S\left(g\right)+3O^{-}\left(ads\right)\to H_{2}O\left(g\right)+SO_{2}\left(g\right)+3e^{-}$$

The high sensitivity of a-IGZO NFs towards H_2_S can be attributed to several factors. First, the morphology of a-IGZO NFs offers a high surface area and abundant adsorption sites for H_2_S gas molecules. It has been reported that in the temperature range from 147 °C to 397 °C, oxygen is dominantly ionosorbed as $O^{-}$ in semiconductor metal oxide materials. This oxygen species plays a critical role in surface reactions, as mentioned above. In this temperature range, the bulk defect effect is slow, and resistance modulation is dominated by the formation and removal of oxygen species [13]. Therefore, the advantage of an improved surface area is important for the H_2_S sensitivity of a-IGZO NFs. Secondly, the amorphous nature of a-IGZO NFs also plays an important role. In the majority of crystallized metal oxide materials, the gas sensitivity depends on the existence of back-to-back Schottky barriers between the domains and the level of gas adsorption across the grain boundaries [24]. If the grain growth becomes too severe, the gas sensitivity can be reduced accordingly. a-IGZO NFs, with their lack of a grain structure, can avoid this issue while facilitating a larger amount of adsorption sites and a lower optimal temperature in comparison to crystalline structures (generally ranging from 300 °C to 450 °C).

Thirdly, the bond dissociation energy of the H-SH bond in H_2_S is relatively small (381 kJ/mol) [12]. This allows H-SH bonds to detach easily and contribute to the surface reaction with NFs. This also facilitates excellent selectivity toward H_2_S. To provide a context for comparison between H_2_S and other gases used in this work, the bond dissociation energies of the mentioned gases are provided in Table S4.

Even though we were unable to detect oxygen vacancies using ex situ XPS, they generally exist in oxide samples. Oxygen vacancies also play an important role in the H_2_S sensing process as they are favorable sites for gas adsorption [55]. These oxygen vacancy defects can serve as sites for oxygen adsorption at the sensing temperature, leading to the higher adsorption of oxygen and, consequently, the occurrence of more sensing reactions between H_2_S and adsorbed oxygen species. Furthermore, three types of cations are present in a-IGZO, namely, In, Ga, and Zn, which can act as adsorption sites for H_2_S gas molecules [56]. Furthermore, Zn also generates more oxygen vacancies, while Ga subdues excessive oxygen defect formation, helping stabilize the oxide matrix and reduce baseline drift [57]. In, with its highly dispersive conduction band, magnifies the sensing signal through enhanced electron mobility [58]. The above reasons contribute to the enhanced sensitivity of a-IGZO toward H_2_S gas.

## 4. Conclusions

In this study, a-IGZO NFs were synthesized by electrospinning followed by subsequent heat treatment at 500 °C to remove the polymeric species, while preserving the amorphous nature of the NFs. The gas sensing performance of a-IGZO NFs was thoroughly investigated. The sensor showed a response of 40.5 to 100 ppm H_2_S gas at 250 °C. Also, it was able to detect as low as 500 ppb H_2_S gas. The sensor also exhibited good stability and repeatability, as well as excellent selectivity. The superior performance was attributed to the formation of amorphous NFs with a large surface area, no grain structure, and a low H_2_S bond dissociation energy, as well as a strong H_2_S chemical interaction. This study demonstrated the strong potential of a-IGZO NFs for H_2_S sensing with high sensitivity and selectivity.

## Figures and Tables

**Table 1:** Comparison of H_2_S gas sensing properties of a-IGZO NF sensor with those of other sensors reported in the literature.

Sensing Material	Conc. (ppm)	Experimental Detection Limit (ppm)	T (°C)	Response (R_a_/R_g_) or (R_g_/R_a_)	Ref.
a-IGZO NFs	100	1	250	40.5	Present Work
ZnO/In_2_O_3_ nanorods	100	10	300	1.1	[50]
Cr_2_O_3_ nano-sized cylinders/ellipsoids	100	0.001	170	42.81	[51]
ZnO hollow nanocages	10	0.1	275	9.08	[52]
B- and N-codoped graphene/mesoporous NiO nanodisks	100	1	50	5.84	[53]
NiO thin films	200	1	400	28.8	[54]

## Data Availability

The data presented in this study are available on request from the corresponding author.

## Supplementary Materials

The following supporting information can be downloaded at: https://www.mdpi.com/article/10.3390/s26061936/s1, Figure S1: XPS survey spectra of IGZO NFs heat-treated at 400 °C, 500 °C and 800 °C; Figure S2: XPS raw and background-corrected spectra of core-regions of (a) In 3d (b) Ga 2p (c) Zn 2p and (d) O 1s with fitted and residual spectra; Figure S3: (a) Dynamic resistance curves for 4 fabricated a-IGZO NFs gas sensors to 100, 50, 10 ppm H_2_S at 250 °C, (b) standard deviation of response versus concentration; Figure S4: Baseline resistance over temperature of the a-IGZO NFs gas sensor; Table S1: Response values of a-IGZO NFs to 100, 50, 10 ppm H_2_S at various temperatures; Table S2: Response values of a-IGZO NFs sensor to different gases with various concentrations at 250 °C; Table S3: Responses of a-IGZO NFs fresh sensor and after-30-day sensor to 10 ppm H_2_S concentration at 250 °C during five cycles; Table S4: Bond energies of the gases used in this study; Table S5: XPS analysis results for atomic percentages of NFs annealed at 400 °C, 500 °C and 800 °C. References [56,59,60,61] are cited in the Supplementary Materials.
