# Supplementary material for: Electrospun Amorphous Indium Gallium Zinc Oxide (IGZO) Nanofibers for Highly Selective H2S Gas Sensing
Source: Sensors (Basel). 2026 Mar 19;26(6):1936. doi: 10.3390/s26061936 (PMC13030730; doi:10.3390/s26061936)
Supplement: Supplementary file 1 [file sensors-26-01936-s001.zip › sensors-4195636-supplementary.pdf]

Supporting Information

Electrospun Amorphous Indium Gallium Zinc Oxide (IGZO) Nanofibers for Highly Selective H_2S Gas Sensing

Anh-Duy Nguyen[1,†], Sung Tae Lim[1,†], Jong Heon Kim[1], Yujin Kim[1], Gayoung Yoon[1], Ali Mirzaei[2], Hyoun Woo Kim[3], Sang Sub Kim[1,*]

[1]Department of Materials Science and Engineering, Inha University, Incheon 22212, South Korea

[2]Department of Materials Science and Engineering, Shiraz University of Technology, Shiraz 715557-13876, Iran

[3]Division of Materials Science and Engineering, Hanyang University, Seoul 04763, South Korea

***Correspondence:** sangsub@inha.ac.kr

† These authors contributed equally to this work.

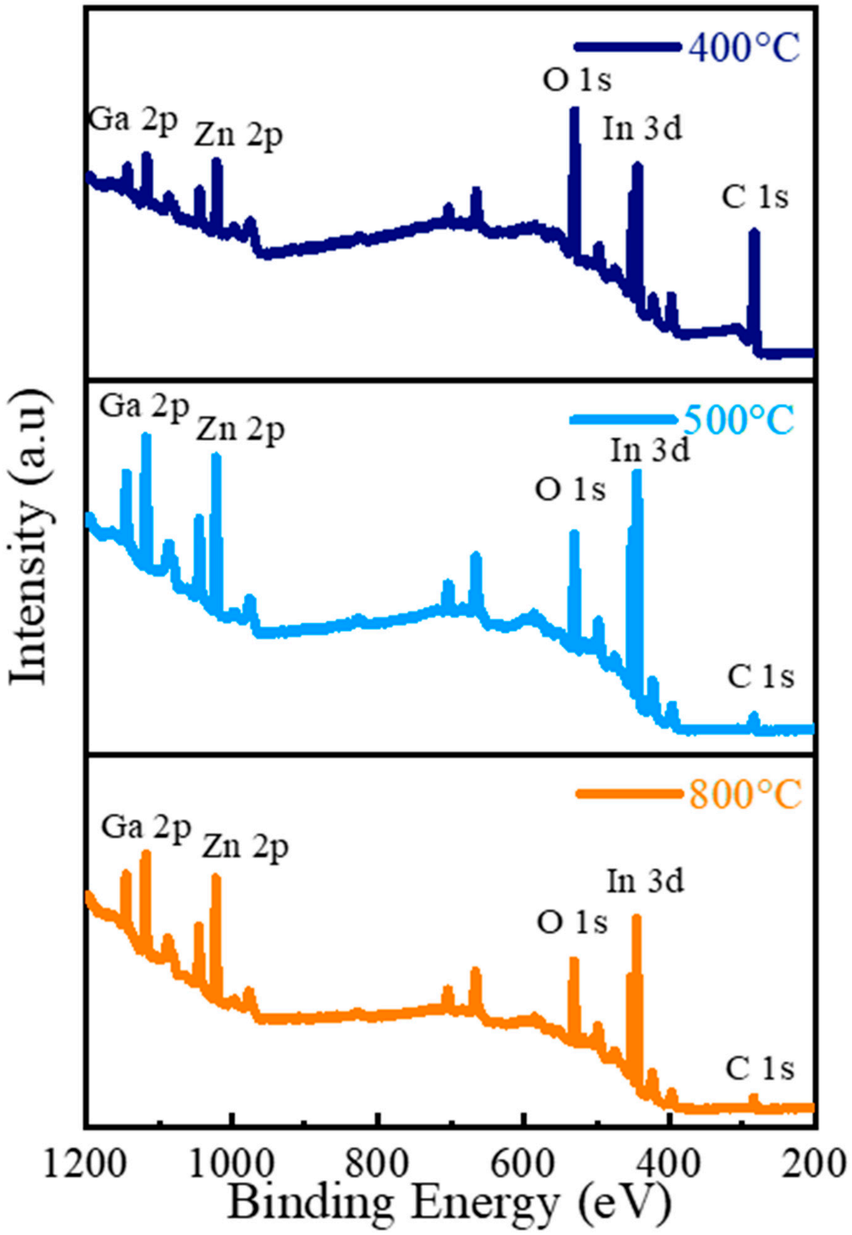

Figure S1. XPS survey spectra of IGZO NFs heat-treated at 400°C, 500°C, and 800°C.

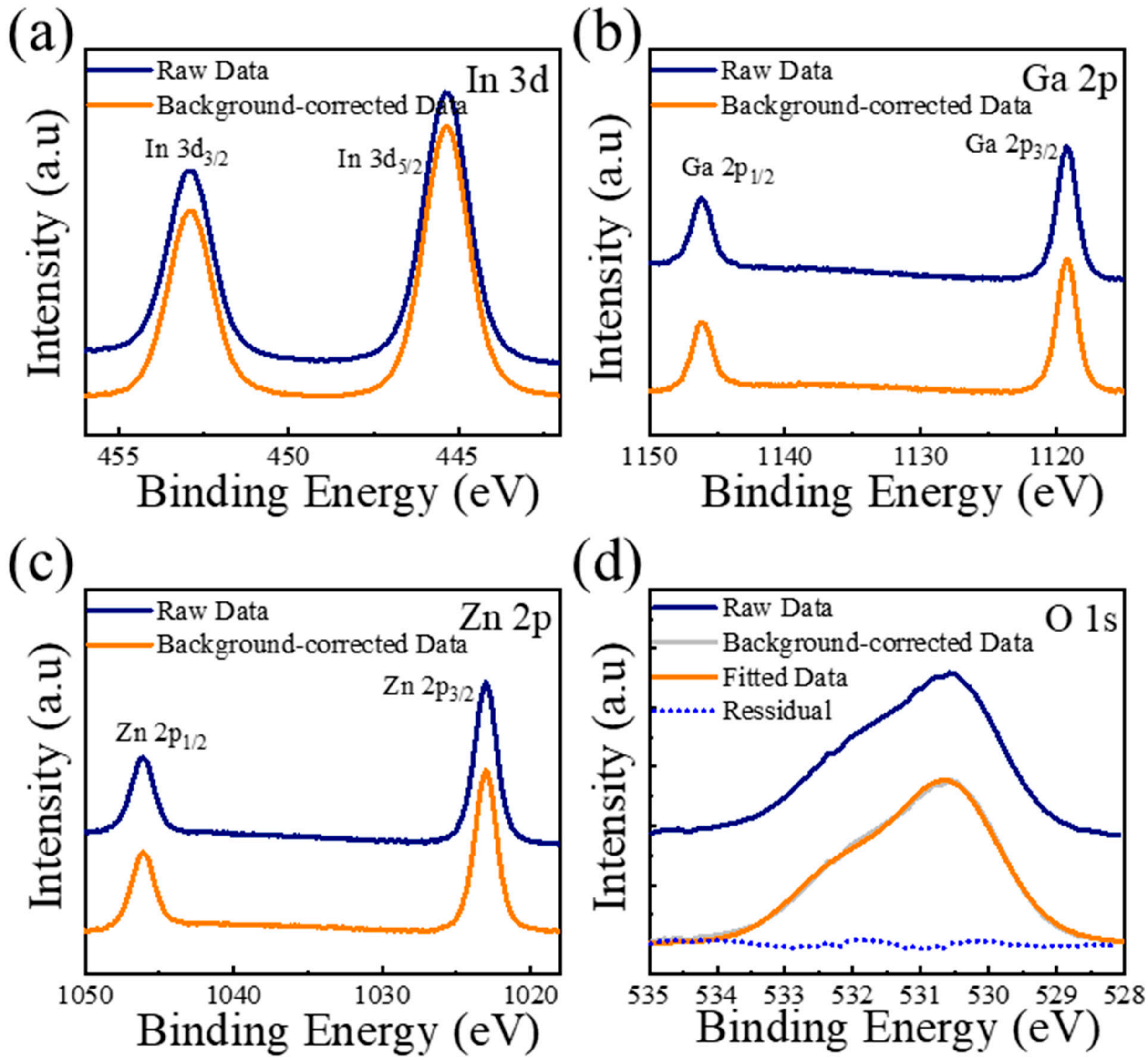

Figure S2. XPS raw and background-corrected spectra of core regions of (a) In 3d, (b) Ga 2p, (c) Zn 2p, and (d) O 1s with fitted and residual spectra.

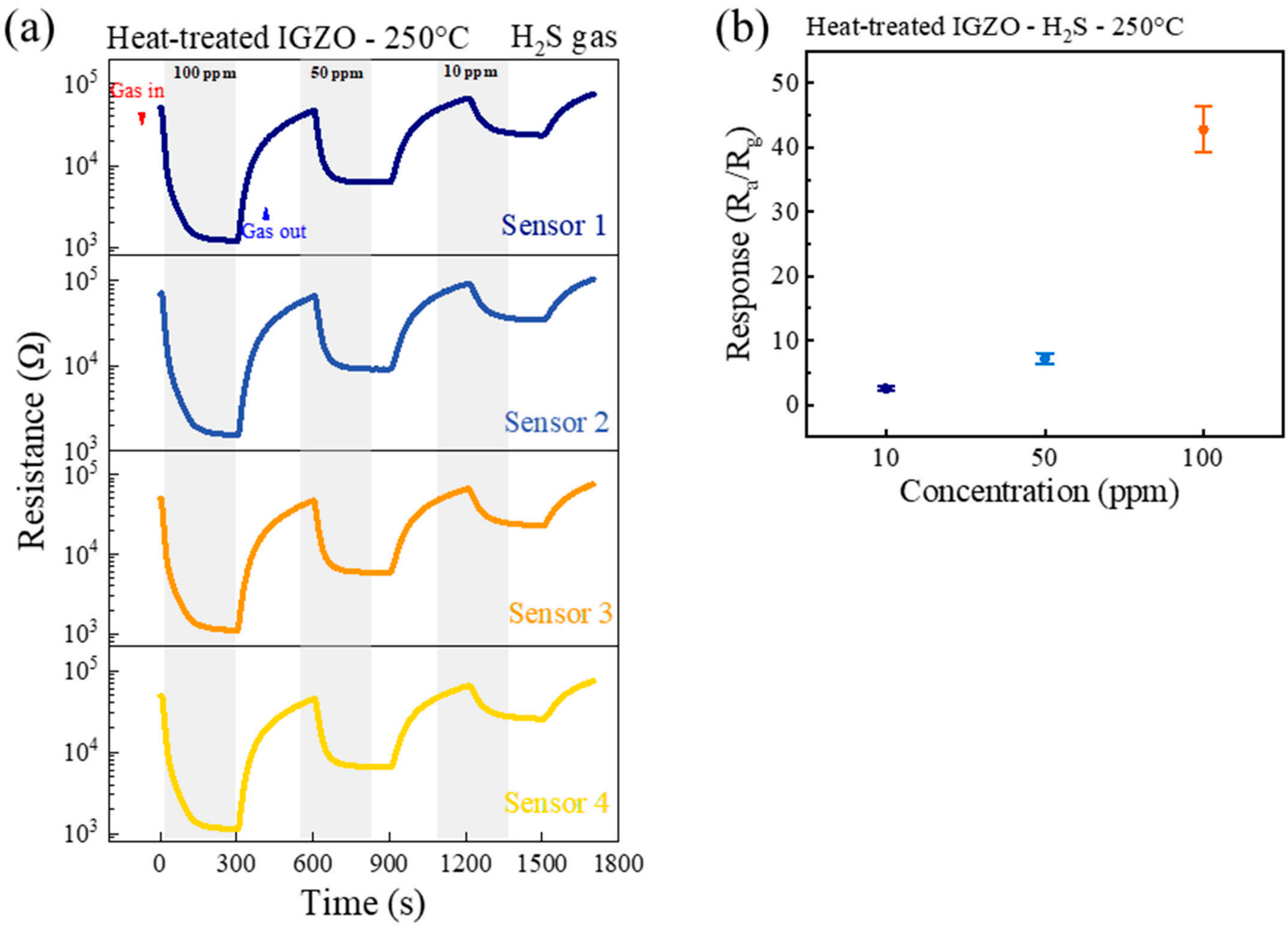

Figure S3. (a) Dynamic resistance curves for 4 fabricated a-IGZO NF gas sensors to 100, 50, and 10 ppm H_2S at 250°C, (b) standard deviation of response versus concentration.

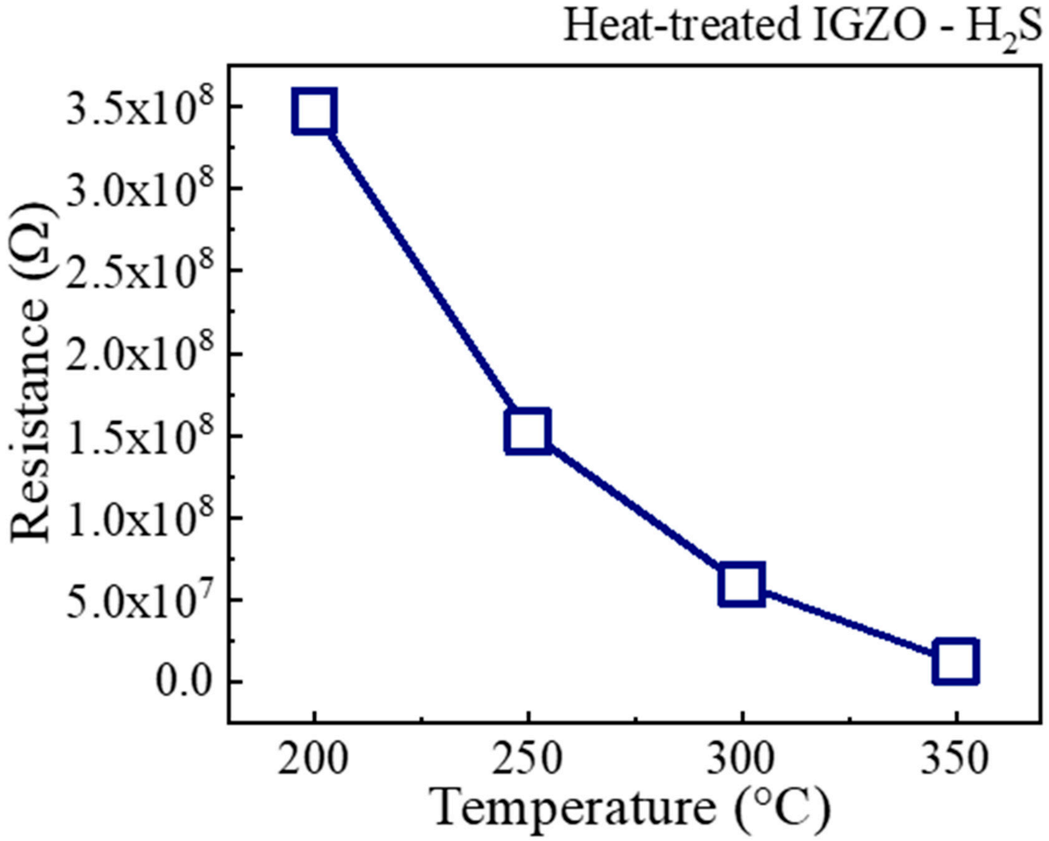

Figure S4. Baseline resistance over temperature of the a-IGZO NF gas sensor.

Table S1. Response values of a-IGZO NFs to 100, 50, and 10 ppm H_2S at various temperatures.

Concentration (ppm)		T (°C)			
		200	250	300	350
**a-IGZO NFs**	10	3.42	5.76	6.43	3.17
	50	93	15.61	15.75	8.52
	100	15.81	40.52	40.12	18.02

Table S2. Response values of a-IGZO NF sensor to different gases with various concentrations at 250°C.

Concentration (ppm)		Gases							
		H_2S	NO_2	C_7H_8	C_3H_6O	C_2H_5OH	C_6H_6	CO	H_2
		Response (R_a/R_g)							
**250°C**	10	5.7	1.06	1.06	1.04	1.07	1.03	1.04	1.02
	50	15.6	1.07	1.17	1.07	1.08	1.04	1.18	1.06
	100	40.5	1.19	1.18	1.11	1.19	1.12	1.71	1.22

Table S3. Responses to 10 ppm H_2S of a-IGZO NF fresh sensor and after-30-day sensor at 250°C during five cycles.

Cycle Number	After 60 days
1	2.31
2	2.48
3	2.67
4	2.84
5	2.93

Table S4. Bond energies of the gases used in this study [1–4].

Gas Type	Bond	Bond energy (kJ/mol)
H_2S	H-SH	381
C_6H_6	H-C_6H_5	472.8
H_2	H-H	436
C_3H_6O	H-C_3H_5O	393
NO_2	O-NO	300
CO	C-O	1076.5
C_7H_8	H-C_6H_7	378.2
C_2H_5OH	H-C_2H_5O	436

Table S5. XPS analysis results for atomic percentages of NFs annealed at 400°C, 500°C, and 800°C.

Element	Annealing Temperature (°C)		
	400	500	800
In	8.64	13.02	14.11
Ga	7.77	15.45	16.48
Zn	8.52	15.33	15.43
O	75.07	56.20	53.98
Total	100	100	100

References

1. Bulemo, P.M.; Kim, D.-H.; Shin, H.; Cho, H.-J.; Koo, W.-T.; Choi, S.-J.; Park, C.; Ahn, J.; Güntner, A.T.; Penner, R.M.; et al. Selectivity in Chemiresistive Gas Sensors: Strategies and Challenges. *Chem. Rev.* 2025, *125*, 4111–4183, doi:10.1021/acs.chemrev.4c00592.
2. Kim, Y.; Lee, J.H.; Kim, J.H.; Shin, R.-H.; Park, J.H.; Mirzaei, A.; Kim, S.S.; Kim, J.-H. Ultrasensitive and Selective CuO/GaN Co-Decorated SnO2 Nanowire Gas Sensor with Ppb-Level Detection of H2S Gas. *Sens. Actuators B Chem.* 2026, *447*, 138879, doi:10.1016/j.snb.2025.138879.
3. Phuoc, P.H.; Hung, C.M.; Van Toan, N.; Van Duy, N.; Hoa, N.D.; Van Hieu, N. One-Step Fabrication of SnO2 Porous Nanofiber Gas Sensors for Sub-Ppm H2S Detection. *Sens. Actuators A Phys.* 2020, *303*, 111722, doi:10.1016/j.sna.2019.111722.
4. Mueller, J.A.; Rogers, S.A.; Houston, P.L. Zero Kinetic Energy Photofragment Spectroscopy: The Threshold Dissociation of NO2. *Journal of Physical Chemistry A* 1998, *102*, 9666–9673, doi:10.1021/jp982634m.
